# Enhancement of mouse hematopoietic stem/progenitor cell function via transient gene delivery using integration-deficient lentiviral vectors

**DOI:** 10.1016/j.exphem.2017.09.003

**Published:** 2018-01

**Authors:** Maria E. Alonso-Ferrero, Niek P. van Til, Kerol Bartolovic, Márcia F. Mata, Gerard Wagemaker, Dale Moulding, David A. Williams, Christine Kinnon, Simon N. Waddington, Michael D. Milsom, Steven J. Howe

**Affiliations:** a Molecular and Cellular Immunology, University College London Great Ormond Street Institute of Child Health, London, UK; b Department of Hematology, Erasmus University Medical Center, Rotterdam, The Netherlands; c Laboratory of Translational Immunology, University Medical Center Utrecht, Utrecht, The Netherlands; d Weatherall Institute of Molecular Medicine, University of Oxford, Oxford, UK; e Department of Bioengineering and Institute for Bioengineering and Biosciences, Instituto Superior Técnico, Universidade de Lisboa, Lisboa, Portugal; f Hacettepe University, Stem Cell Research and Development Center, Ankara, Turkey; g Raisa Gorbacheva Memorial Research Institute for Pediatric Oncology and Hematology, Saint Petersburg, Russia; h Boston Children's Hospital, Harvard Medical School, Boston, MA, USA; i Gene Transfer Technology Group, University College London Institute for Women's Health, London, UK; j Antiviral Gene Therapy Research Unit, Faculty of Health Sciences, University of the Witwatersrand, Johannesburg, South Africa; k Experimental Hematology Group, Heidelberg Institute for Stem Cell Technology and Experimental Medicine (HI-STEM) and the German Cancer Research Center, Im Neuenheimer Feld 280, Heidelberg, Germany

## Abstract

Integration-deficient lentiviruses (IdLVs) deliver genes effectively to tissues but are lost rapidly from dividing cells. This property can be harnessed to express transgenes transiently to manipulate cell biology. Here, we demonstrate the utility of short-term gene expression to improve functional potency of hematopoietic stem and progenitor cells (HSPCs) during transplantation by delivering *HOXB4* and *Angptl3* using IdLVs to enhance the engraftment of HSPCs. Constitutive overexpression of either of these genes is likely to be undesirable, but the transient nature of IdLVs reduces this risk and those associated with unsolicited gene expression in daughter cells. Transient expression led to increased multilineage hematopoietic engraftment in in vivo competitive repopulation assays without the side effects reported in constitutive overexpression models. Adult stem cell fate has not been programmed previously using IdLVs, but we demonstrate that these transient gene expression tools can produce clinically relevant alterations or be applied to investigate basic biology.

## Highlights

• Integration-deficient vectors (IdLVs) express genes transiently in dividing stem cells.
• Hematopoietic stem/progenitor cells (HSPCs) can be programmed using IdLVs.
• HOXB4 or Angptl3 expression from IdLVs improves engraftment of transplanted HSPCs.
• Short-term gene delivery avoids the side effects associated with constitutive expression.

Improved tissue engineering and reprogramming of adult and pluripotent stem cells could be achieved through controlled, time-restricted gene expression. Application of transcription factors and other genes involved in cell fate decisions often requires transient signals that are difficult to control with current technologies without applying exogenous chemicals to induce or repress promoter activity. Here, we demonstrate the utility of short-term gene expression from a viral delivery vector to alter progenitor cell behavior using hematopoietic stem and progenitor cells (HSPCs) as a model system.

Inherited and acquired blood disorders are treated by HSPC transplantation. Altering cellular characteristics, such as replication, homing, and engraftment, could improve clinical outcome; the ability to expand cell populations would be beneficial, especially when the number of donor HSPCs is limited. Many genes have been proposed to assist expansion of cell populations while maintaining the progenitor pool, but long-term overexpression could be detrimental [1]. Lentiviral vectors can deliver genes efficiently to a large range of cells for biological experimentation or for gene therapy [2], where they are showing promise in clinical trials [3]. However, conventional lentiviral vectors integrate their transgene payload permanently into the host cell's genome, which is not desirable in the setting of transient cell fate programming. Integration-deficient lentiviral vectors (IdLVs) deliver a genetic payload, but, due to mutations within the viral integrase gene [4], cannot mediate stable integration of the reverse-transcribed proviral DNA into the host cells' chromosomes. In dividing cells, this results in dilution of episomal DNA with expression of the transgene rapidly falling to undetectable levels [5].

Here, the ability of IdLVs to influence HPSC behavior was assessed in HSPCs using transplantation as the functional read-out. These vectors were used for the transient expression of two genes involved in blood progenitor cell maintenance and expansion, human *Homeobox B4* (*HOXB4*) and murine *Angiopoietin-like 3* (*Angptl3*), and to compare them with standard, integration-proficient lentiviral vectors (IpLVs). HOXB4 has been reported previously to maintain stem cell function during cell expansion 6, 7 and was therefore applied using IdLVs to test whether this approach can facilitate biologically relevant changes in cells without the drawbacks of integrating vectors [8] or the need for exogenous drugs to control promoter activity. Although it is unlikely that *HOXB4* would be useful in a clinical setting, the application of the protein and the resulting effect on HSPC expansion is well understood, making it useful for these proof-of-concept experiments. *Angptl3* is less characterized, but it has shown promise in expanding HSPC populations when applied as a protein or permanently expressed in cells 9, 10, 11; therefore, it was also delivered using IdLVs to measure the biological effects of its short-term expression in cells.

## Methods

### Virus production

Vesicular stomatis virus G (VSV-G)-pseudotyped pLBid.nlsCre.SF.mCherry [12], pRRL.PPT.SF.co-HOXB4.bPRE4*, and pRRL.PPT.SF.co-Angptl3.bPRE4* vectors were produced using second-generation packaging plasmids as described previously [13] both with and without the D64V integrase mutation [4] to package IdLVs and IpLVs, respectively. IpLVs expressing a *Venus* reporter gene were used as control vectors.

### LSK cell isolation and growth

HSPC Lin^−^ Sca-1^+^ C-kit^+^ (LSK) cells were separated after isolation of bone marrow (BM) cells by flushing mouse femur and tibia bones. Harvested cells were stained with the lineage antigens CD3, CD45R (B220), CD11b, Gr-1 (Ly-6G/C), 7–4, and Ter-119. Lin^−^ cells were isolated using the mouse Lineage Cell Depletion Kit (Miltenyi Biotec) following the manufacturer's recommendations. Lin^−^ isolated cells were stained with streptavidin-fluorescein isothiocyanate (FITC), phycoerythrin (PE) Ly-6A/E (Sca-1), and allophycocyanin (APC) CD117 (c-kit) (all obtained from BD Biosciences), and the LSK population was isolated in a **MoFlo XDP** sorter (Beckman Coulter) (gating strategy shown in Supplementary Figure E1, online only, available at www.exphem.org). Cells were resuspended at 5 × 10^5^ cells/mL in StemSpan SFEM (StemCell Technologies), supplemented with 0.5% penicillin–streptomycin, murine stem cell factor (mSCF; 300 ng/mL), human thrombopoietin (hTPO; 100 ng/mL, R&D Systems), and FLT3-L (100 ng/mL; Miltenyi Biotec). After 12 hours of prestimulation at 37°C, 5% CO_2_ fully humidified air, LSKs were transduced with vectors at various multiplicities of infection (MOIs) for 24 hours by adding virus to the medium.

### *In vitro* colony-forming unit assays

A total of 100 LSKs were seeded in MethoCult GF-M3534 medium (StemCell Technologies) in triplicate in 35-mm plastic plates and cultured at 37°C, 5% CO_2_, fully humidified air. After 7 days, colony numbers were scored.

### *In vitro* cre-*loxP* experiments

K562 cells were transduced with SF91.LoxP.MCSI.eGFP.LoxP.EBFP2.bPre vector particles at low MOI, aiming for one vector copy per cell (<10% green fluorescent protein-positive [GFP^+^]). These cells were expanded, sorted for GFP fluorescence, and serially diluted to select clones. This target cell line was then transduced with IpLV or IdLV pLBid.nlsCre.SF.mCherry and levels of enhanced GFP (eGFP), blue fluorescent protein (BFP), and mCherry measured by flow cytometry over time.

### *In vivo* cre-*loxP* experiments

Donor LSK cells were isolated from ROSA26-eYFP mouse BM. After transduction (MOI 100), 2 × 10^4^ CD45.1-LSK cells were injected into the tail veins of lethally irradiated C56BL/6J recipients (6 Gy/4 Gy split-dose over 2 days). Results were obtained from three mock-transduced mice or groups of four mice receiving IpLV- or IdLV-transduced cells.

### Reverse transcriptase polymerase chain reaction

Levels of HOXB4 were measured by isolating RNA from 1 × 10^4^ Lin^−^ cells (Qiagen, RNEasy minikit). RNA was treated with DNAse then reverse transcribed. Reverse transcriptase polymerase chain reaction (RT-PCR) for HOXB4 or a gapdh control was performed using the Applied Biosystems gapdh primer set Mm99999915_g1 or the following primers specific to PRE element present in the 3′ untranslated region of the HOXB4 RNA: 5′-TGTGTTTGCTGACGCAACC-3′ and 5′-CCGACAACACCACGGAATT-3′ with cycling conditions 95°C for 15 seconds at 60°C for 1 minute for 40 cycles.

### Vector-specific PCR and titration of virus preparations

DNA from different organs was extracted using a DNeasy Blood and Tissue kit (Qiagen). Titer and vector copy number (VCN) were calculated by real-time PCR (ABI Prism 7000) with primer/probe combinations to *WPRE* and *titin* [14] or to the long terminal repeat: U5-F-5′-TCTGGCTAACTAGGGAACCCA-3′ and U5-R-5′-CTGACTAAAAGGGTCTGAGG-3′ with SYBR green using cycling conditions as above. Alternatively, the same primers were used in a standard PCR reaction and the products separated by electrophoresis in a 2% agarose gel.

Statistical analyses were performed using GraphPad Prism software.

### *In vivo* competitive repopulation assay

**Figure 1:**
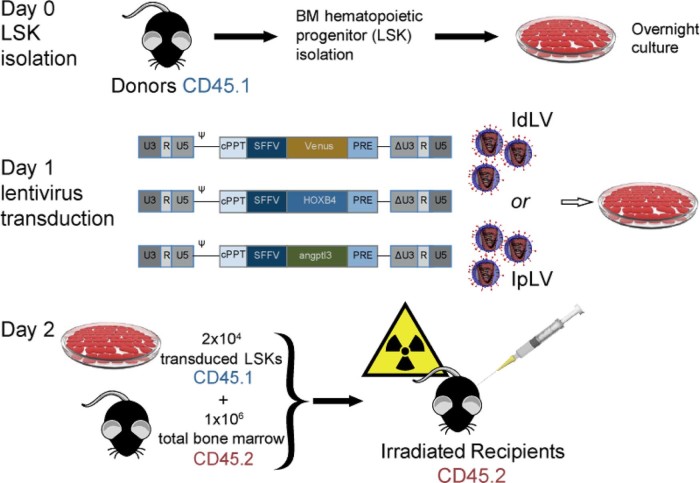
The in vivo competitive repopulation assay. BM cells were obtained by flushing femurs and tibias of C57BL/6J CD45.1 mice before staining with the lineage antigens CD3, CD45R (B220), CD11b, Gr-1 (Ly-6G/C), 7–4, and Ter-119. Lin^−^ cells were isolated using the mouse Lineage Cell Depletion Kit (Miltenyi Biotec) following the manufacturer's recommendations. Lin^−^ isolated cells were stained with streptavidin-FITC, PE Ly-6A/E (Sca-1), and APC CD117 (c-kit) (all obtained from BD Biosciences), and the LSK population was isolated in a **MoFlo XDP** sorter (Beckman Coulter). Cells were resuspended at 5 × 10^5^ cells/mL in StemSpan SFEM (StemCell Technologies) supplemented with 0.5% penicillin–streptomycin, mSCF (300 ng/mL), hTPO (100 ng/mL, R&D Systems), and FLT3-L (100 ng/mL; Miltenyi Biotec) and cultured overnight before and after overnight culture and transduced for 24 hours with IdLVs or IpLVs at different MOIs. IdLV or IpLV versions of lentiviral vectors expressing a HOXB4 or Angptl3 or IpLV fluorophore (Venus, control) were used in this study. Self-inactivating vectors were produced with a ΔU3-deleted 3′ long-terminal repeat, with the transgene under the control of the internal spleen focus-forming virus long-terminal repeat promoter element. Transduced LSK cells were harvested the next day, and 2 × 10^4^ of them were injected into the tail veins of lethally irradiated C57BL/6J CD45.2 recipient mice (split dose of 6 Gy and 4 Gy over 2 consecutive days, gamma irradiated using a caesium-137 source, dose rate 3 Gy/minute). One million freshly harvested CD45.2 total BM cells were injected alongside the CD45.1 donor LSKs as competitors. cPPT = Central polypurine tract; IRES = internal ribosomal entry site; PRE = attenuated post-transcriptional regulatory element; U5, R, and (Δ)U3 = (self-inactivating) long-terminal repeats; Ψ = packaging signal.

C57BL/6J(CD45.2) mice were used as recipients and as a source of BM competitor cells and B6.SJL-PtprcaPep3b/BoyJ(CD45.1) mice as LSK cell donors (Fig. 1). After transduction, 2 × 10^4^ CD45.1-LSK cells and 10^6^ freshly harvested CD45.2 total BM cells were injected into the tail veins of lethally irradiated recipients (6 Gy/4 Gy split-dose over 2 days). Results were obtained from four separate reconstitution assays with the following numbers of animals in each group in total: Venus-IpLV100, *n* = 7; HOXB4-IdLV200, *n* = 6; HOXB4-IdLV500, *n* = 6; HOXB4-IpLV100, *n* = 8; Angptl3-IdLV500, *n* = 3; and Angptl3-IpLV100, *n* = 7.

The Institutional Research Ethics Committee approved all animal procedures (Institute of Child Health, UCL) performed under UK Home Office License PPL70/7024.

### Flow cytometry

Chimerism of CD45.1 cells in peripheral blood (30, 60, and 90 days after transplantation) and of BM and spleen collected at the end of the 90-day experiment were analysed by flow cytometry (LSRII, BDBiosciences) with antibodies targeting CD45.1 antigen combined with markers for different blood cell lineages. The same machine was used to measure expression of enhanced yellow fluorescent protein (eYFP), eGFP, BFP, and mCherry using untransduced cells to set the gating strategy.

## Results

The expression kinetics of genes delivered with IdLVs and IpLVs were determined in vitro in an immortalized K562 cell model. A gamma-retroviral vector, SF91 loxP MCSI eGFP loxP eBFPco bPRE, was used to insert a construct containing an eGFP marker gene flanked by *loxP* sites and was excised when cre recombinase was expressed in the cells, concurrently initiating expression of a downstream BFP. IpLVs and IdLVs were used to deliver the *cre* recombinase gene coexpressed from a bidirectional promoter with *mCherry* fluorescent protein [12] (Supplementary Figure E2A, online only, available at www.exphem.org). Delivery of cre recombinase with either vector at increasing MOIs reduced eGFP expression progressively over time while BFP expression commenced. The lack of detectable mCherry expression from IdLVs after 4 days demonstrated the transient nature of the gene delivery to these dividing cells, but short-term expression of cre produced a permanent alteration in eGFP/BFP production. The same result was achieved with integrating IpLVs, in which expression of cre/mcherry was stable over the 10-day period (Supplementary Figure E2B, online only, available at www.exphem.org). The use of a higher MOI (50) of IpLVs appeared to be toxic (data not shown), probably due to higher expression levels from IpLVs [15].

**Figure 2:**
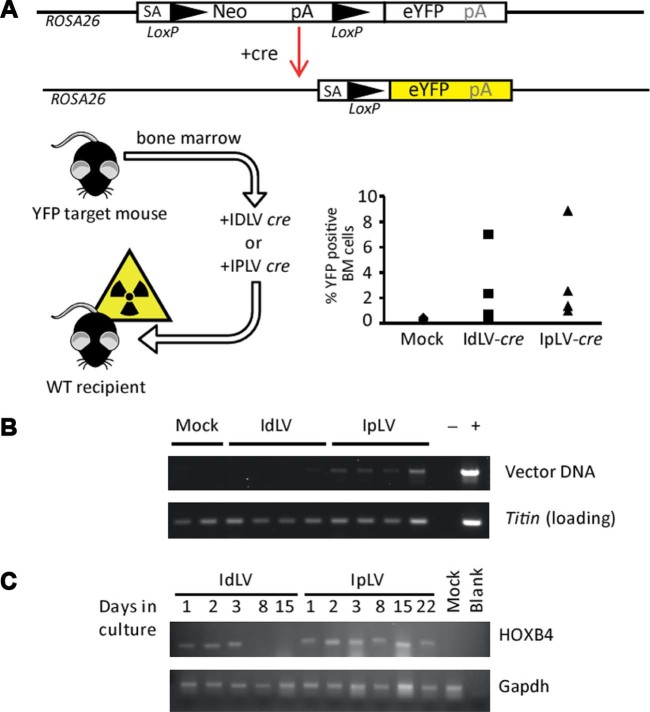
IdLV transduction of BM cells provides transient gene expression in vitro and in vivo. LSK cells were isolated from BM harvested from *ROSA26*-eYFP mice that contain an eYFP gene at the *ROSA26* locus that is expressed upon exposure to cre-recombinase, which removes a disruptive neomycin (Neo) cassette [16]. Cells were transduced with IdLVs or IpLVs encoding cre recombinase at an MOI of 100 before injecting into lethally irradiated wild-type (YFP-negative) recipients. **(A)** Analysis of recipient BM 6 months later revealed YFP-positive cells engrafted after cre recombinase expression from IdLVs and IpLVs. **(B)** Vector-specific PCR confirmed the long-term persistence for vector in IpLV samples, but not IdLV. **(C)** Lin^−^ cells were transduced with IdLVs or IpLVs expressing HOXB4 and grown in liquid culture. RT-PCR showed that HOXB4 expression from IdLVs can be observed for up to 3 days when cells are in culture, whereas IpLVs expressed the gene until the end of the experiment (22 days). Primers specific for gapdh were used as a positive control for the RT-PCR. blank = Non-template PCR control; mock = mock-transduced cells.

To determine whether a brief pulse of expression could produce an equivalent permanent change in vivo, a similar system in a mouse model in which cre recombinase induces eYFP expression in cells was tested [16]. Transduction of LSK BM cells from target mice with IpLVs or IdLVs expressing *cre* before transplantation into YFP-negative recipient mice gave rise to expression of eYFP-positive cells in the BM space (Fig. 2A), whereas the YFP locus in mock-transduced donor cells was not activated, and the marker gene was not observed in recipients. After 6 months, the *cre* gene delivered by IpLVs was maintained in the host cells' genome, but IdLV was not detectable by PCR in three of four mice. The very faint signal in the fourth mouse reveals a possible background integration event or PCR contamination (Fig. 2B).

With the cre-lox experiments providing clear evidence that transduction of cells with IdLVs can result in measurable changes in cells, the system was tested in mouse Lin^−^ BM HSPCs to observe the expression kinetics of the HOXB4 transcription factor. Levels of *HOXB4* RNA reduced to background signal in cells transduced with IdLV after 3 days growing in liquid culture. *HOXB4* RNA was produced from the IpLVs until the last time-point after 22 days in culture, confirming the limited duration of expression from IdLV in HSPC (Fig. 2C), compared with standard integrating vectors.

LSK HSPC cells were harvested from mouse BM and transduced with IpLVs and IdLVs to deliver *HOXB4* at different MOIs to determine whether transient expression of the gene can maintain or expand HSPC in vitro. In most cases, this generated limited expansion of progenitor cells from mouse LSK populations as expected (Supplementary Figure E3A, online only, available at www.exphem.org) [17]. However, when grown in liquid culture, which normally induces HSPC differentiation and reduces self-renewal activity [18], IdLV-mediated delivery of HOXB4 and Angptl3 resulted in a trend toward enhanced maintenance of differentiation potential when cultured over 7 days and tested in clonogenic assays compared with controls (Supplementary Figure E3B, online only, available at www.exphem.org). Despite inherent assay variability, IdLV produced similar biological effects to IpLV, warranting further testing in vivo.

LSK cells from congenic C57BL/6J CD45.1^+^ mice were isolated to high purity (Supplementary Figure E1, online only, available at www.exphem.org) and transduced with IpLV and IdLV to deliver genes encoding *HOXB4* or *Angptl3* or a *Venus* IpLV fluorophore gene control at MOIs between 100 and 500 (based on PCR, not a functional titer). IdLV transcriptional activity was lower than that for IpLV (data not shown and as reported previously [15]), so increased IdLV concentrations were used to compensate [19].

**Figure 3:**
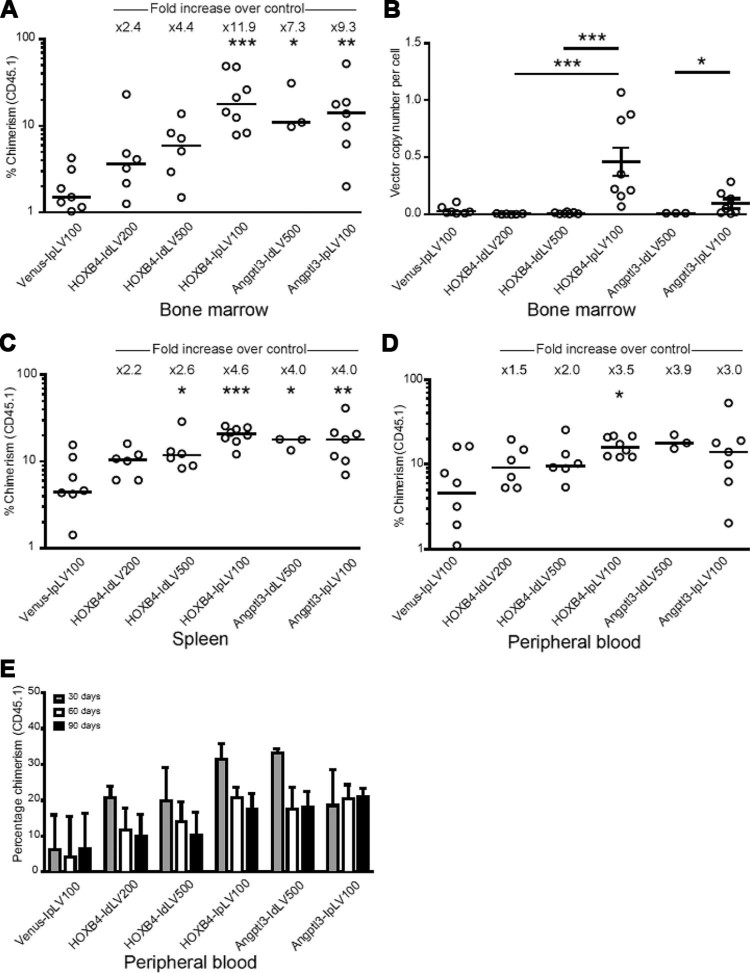
Transient delivery of HOXB4 and Angptl3 increases long-term donor cell chimerism in BM, spleen, and peripheral blood cells. IpLVs or IdLVs expressing HOXB4 or Angptl3 at different MOIs (100–500) or a control IpLV expressing the fluorophore Venus were used to transduce CD45.1 donor cells, which were transplanted into CD45.2 mice in an in vivo competitive repopulation assay. **(A)** BM cells from CD45.2 recipients were analyzed by flow cytometry to determine chimerism of donor CD45.1 cells at 90 days after injection. There was a statistical difference between samples over the whole group for BM (*p* < 0.0001, percentage data normalized by inverse-sine transformation followed by one-way analysis of variance [ANOVA]). *p < 0.5, **p < 0.01, ***p < 0.001 (Šidák's post hoc multiple comparison test vs. Venus IpLV 100). Bar shows the median value with fold increase in median values of samples compared with the control (top). **(B)** Real-time PCR vector copy number analysis in BM cells after 90 days. Error bars indicate mean value ±95% confidence intervals. Overall *p* < 0.0001, one-way ANOVA of copy numbers normalized by log transformation; *p < 0.5, ***p < 0.001, Šidák's post hoc multiple-comparisons test for comparisons between HOXB4 vectors and, separately, between Angptl3-treated mice. **(C)** Spleen and **(D)** peripheral blood from CD45.2 recipients were analyzed by flow cytometry to determine chimerism of transduced donor cells. There was a statistical difference between samples over the whole group for spleen cells collected at 90 days, *p* < 0.0001. Differences in chimerism in peripheral blood between groups did not reach statistical significance, except for HOXB4-IpLV100 versus Venus-IpLV100 (percentage data normalized by inverse-sine transformation followed by one-way ANOVA). *p < 0.5, **p < 0.01, ***p < 0.001, Šidák's post hoc multiple-comparisons test versus Venus-IpLV 100. **(E)** CD45.1 chimerism in the peripheral blood was measured at three time points over the 90-day period. Data shown are the median value; error bars indicate interquartile range.

Using a competitive repopulation assay to determine the engraftment potential and fitness of cultured HSPCs (outlined in Fig. 1), ectopic expression of HOXB4 or Angptl3 improved engraftment of CD45.1 donor cells in BM compared with Venus controls (Fig. 3A). Constitutive overexpression of HOXB4 (HOXB4-IpLV) resulted in the greatest increase in chimerism of donor cells (11.9-fold median increase over Venus transduction, *p* < 0.001). Transient HOXB4 expression from IdLV resulted in a visible trend toward increased engraftment, but did not reach statistical significance (2.4- and 3.4-fold median increase in engraftment at an HOXB4-IdLV[MOI] 200 and 500, respectively). However, both the integrating and versions of the *Angptl3*-containing vectors were able to increase HSPC engraftment significantly, resulting in 7.3- and 9.3-fold increased levels of donor chimerism over controls for IdLVs and IpLVs, respectively (*p* < 0.05, *p* < 0.01, Fig. 3A). VCN analysis in BM cells (Fig. 3B) showed that IpLV proviral DNA was integrated and maintained in BM as expected, whereas IdLV was undetectable or present in negligible amounts after 90 days, despite using higher MOIs (reduction in VCN vs. IpLV: 56- or 60-fold [HOXB4-IdLV200/500, *p* < 0.001] or 13-fold [Angptl3-IdLV500, *p* < 0.05]). Similar improvements in engraftment were observed in the spleen and peripheral blood of these mice (Fig. 3C and 3D).

Peripheral blood from these transplanted animals was monitored over 3 months, with results mirroring engraftment patterns in BM (Fig. 3E). Stable engraftment was apparent in all samples, with *Angptl3*-transduced samples showing the least decline in chimerism.

**Figure 4:**
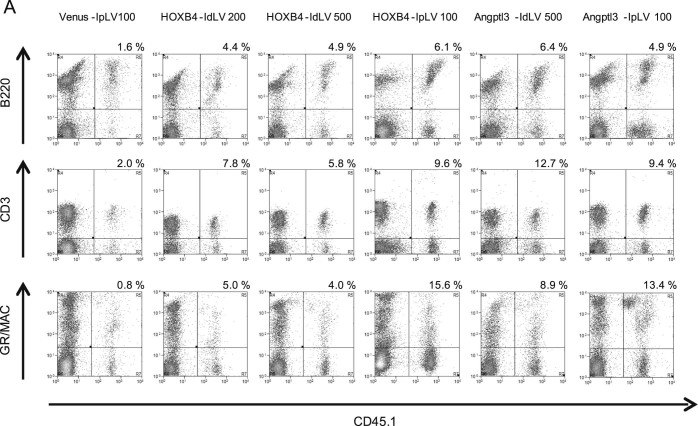

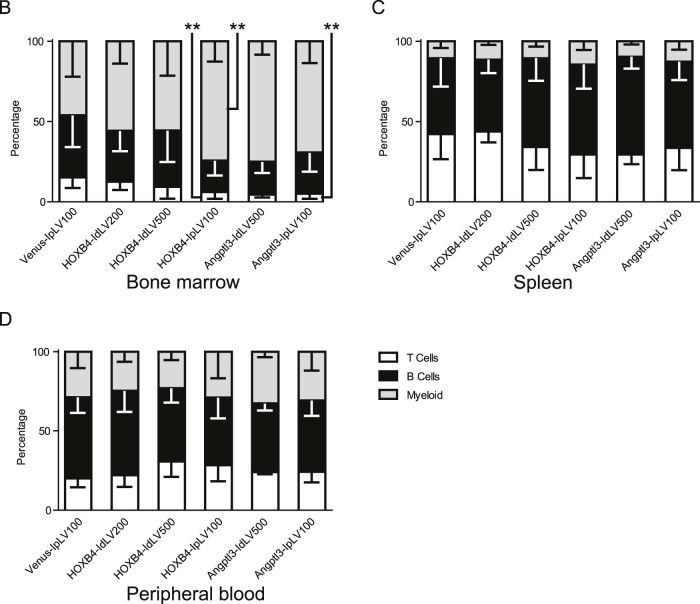
Expression of HOXB4 or Angptl3 from IdLV does not skew lineage differentiation in transduced cells. **(A)** Representative flow cytometry plots showing levels of CD45.1 donor-derived T (CD3), B (B220), and myeloid (GR/MAC) cells (upper right quadrant) measured in recipient mouse PBMCs at 90 days after transplantation. The proportion that each lineage contributes to the average total CD45.1 cell population in each group was calculated by flow cytometry analysis in cells collected from **(B)** BM, **(C)** spleen, and **(D)** peripheral blood. One-way ANOVA on inverse sine-transformed percentage data for each lineage within each tissue reached significance (*p* < 0.05) for T-cell and myeloid lineages within BM cells. HOXB4-IpLV100 samples were significantly different from Venus IpLV-100 samples (*p* < 0.01) in the myeloid and T-cell lineages, and Angptl3-IpLV100 had a significantly lower proportion of T cells than the control (*p* < 0.01, Dunnett's post hoc multiple-comparisons test using a 99% confidence interval). *n* ≥ 3 in each group. Error bars indicate standard deviation.

Compared with controls, increased levels of CD45.1 donor cells were observed across multiple hematopoietic lineages (T, B, and myeloid cells) in the peripheral blood 3 months after transplantation, demonstrating that multipotent HSPCs were transduced equally by IpLVs and IdLVs (Fig. 4A).

Constitutive overexpression of HOXB4 has been shown previously to perturb HSPC differentiation toward the myeloid lineages 20, 21. Lineage distribution of the graft within BM of IpLV-HOXB4 and IpLV-Angptl3 mice was altered significantly compared with controls (*p* < 0.01), but, importantly, this aberrant differentiation was rescued in IdLV-transduced grafts (Fig. 4B). T-, B-, or myeloid cell lineage skewing was not observed in the spleen or peripheral blood with any vector (Fig. 4C and 4D).

IdLV-mediated delivery of HOXB4 and Angptl3 into HSPCs appeared to have no permanent detrimental effects upon the differentiation capacity of these cells but, particularly in the case of transient delivery of Angptl3, was sufficient to program HSPC biology to elicit enhanced engraftment capacity in a clinically relevant transplantation model.

## Discussion

Integration-deficient lentiviral vectors can be used to express a gene in dividing cells, including HSPCs, for less than a week, as demonstrated by in vitro and in vivo experiments. This is sufficient to produce a clear biological response to transient expression of cre recombinase as well as to transcription factors.

Transient gene delivery of *HOXB4* and *Angptl3* to HSPC using IdLVs resulted in improved multi-organ engraftment of the LSK cells, demonstrating that short-term expression of these genes can be protective of the pro-differentiation effect of culturing HSPCs. When expressing Angptl3, IdLV produced similar benefits as the integrating vector, albeit at a higher MOI, potentially due to the secreted nature of the protein promoting more widespread effects [10]. Interestingly, *Angptl3* expression elicited greater chimerism of treated cells in this assay than the more extensively tested *HOXB4* gene. This confirms the potential of *Angptl3* for HSPC expansion and the need for greater understanding of this protein and continued testing of the angiopoietin-like gene family 22, 23, 24. However, the increasing trend toward enhanced HSPC engraftment observed with short-term *HOXB4* expression is consistent with the protein's dose-dependent mode of action [25], and the optimal MOI may not have been reached. Although not statistically significant, a reproducible twofold increase in cell numbers would be clinically relevant.

Post-transplantation reconstitution kinetics can be critical for survival, with the aim of minimizing the period of time in which a patient is immunocompromised and susceptible to bleeding and anemia. Early and late progenitor compartments are likely responsible for driving early waves of hematopoietic reconstitution, and the goal was to understand the overall effect on the whole LSK population including these and, potentially, long-term repopulating cells to improve BM cell engraftment after transplantation. The experiments described here were not designed to characterize which cells within the transduced LSK population were modified by IdLV transduction. Transient gene expression was shown to improve total HSPC engraftment without the relative individual contributions of different progenitor populations.

A pulse of transgene expression from IdLV was therefore sufficient to produce biologically relevant changes in stem cells, but with greatly reduced risk of insertional mutagenesis that has caused serious adverse events in gene therapy clinical trials using IpLVs and gamma-retroviral vectors 26, 27, 28. Aberrant experimental results or clinical toxicity associated with high levels of permanent transgene expression and protein production from integrating vectors could also be avoided with IdLVs. Low-level random integration can occur in IdLVs, but at rates approaching four logs lower than integrase-mediated insertions from standard retroviruses 29, 30, 31, and does not always result in a functional gene being integrated.

Long-term overexpression of HOXB4 in HSPCs skews commitment to different lineages and is associated with progression to leukemia [8] Oncogenesis was not observed in any mice, but lineage distribution of the graft within BM of IpLV-HOXB4 and IpLV-Angptl3 mice was altered significantly compared with controls (*p* < 0.01); differences observed in IdLV-treated mice were not statistically significant. This side effect was not evident in IdLV-transduced grafts demonstrating their utility for time-limited expression of genes associated with toxicity without resorting to inducible promoters or similar extrinsic control systems that are often difficult to control robustly in vivo.

## Conclusion

IdLVs provide a platform for short-term gene expression, shown here to be sufficient to influence HSPC characteristics. Such transient expression in any dividing cell type could be applied in many situations in stem cells that require delivery of RNAi constructs, such as “suicide” genes, recombinases, gene-editing tools, and transcription factors 32, 33. IdLVs could therefore be used to affect stem cell fate through reprogramming, direct programming, facilitating differentiation, or expanding cells, with minimal long-term impact on the genome of the target cell or constitutive signaling.

Other methods also can provide transient protein production in this setting 34, 35, 36, 37, 38, but the advantage of IdLVs lies in the efficiency with which they can deliver genes to most cell types, including many sources of stem cells that divide slowly during the initial transduction.

## Supplementary data

Appendix S1 Supplementary Figures E1–E3. Appendix S1
