# Supplementary material for: Enhancement of mouse hematopoietic stem/progenitor cell function via transient gene delivery using integration-deficient lentiviral vectors
Source: Exp Hematol. 2018 Jan;57:21–9. doi: 10.1016/j.exphem.2017.09.003 (PMC5731634; doi:10.1016/j.exphem.2017.09.003)
Supplement: Appendix S1 — Supplementary Figures E1–E3. [file mmc1.docx]

**Supplementary Figures.**

**S1**


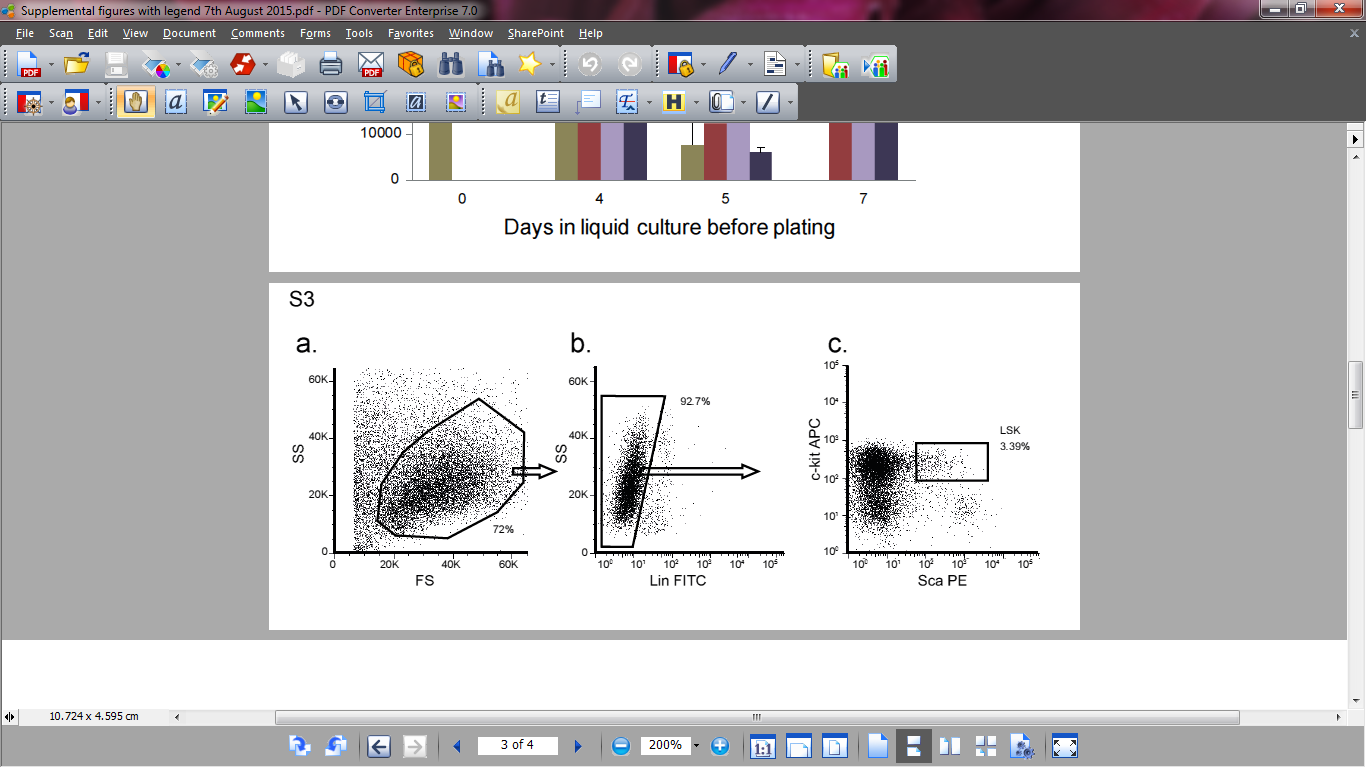


**S1. Gating strategy for isolation of LSK cells by FACS**

The Lineage negative population was isolated from mouse bone marrow using immunomagnetic selection. The cells obtained after the negative selection were stained with streptavidin-FITC, to detect and discard lineage positive cells after the selection with the microbeads, panel b (using a purity of >90% within a live cell gate determined by forward and side scatter (panel a)). Lin^-^ cells were then further purified to select cells that were Sca1^+^ and c-kit^+^ using anti Sca1-PE and anti c-kit-APC (c). The double-positive cells inside the lineage negative population were sorted and used for subsequent *in vitro* and *in vivo* experiments.

**S2**


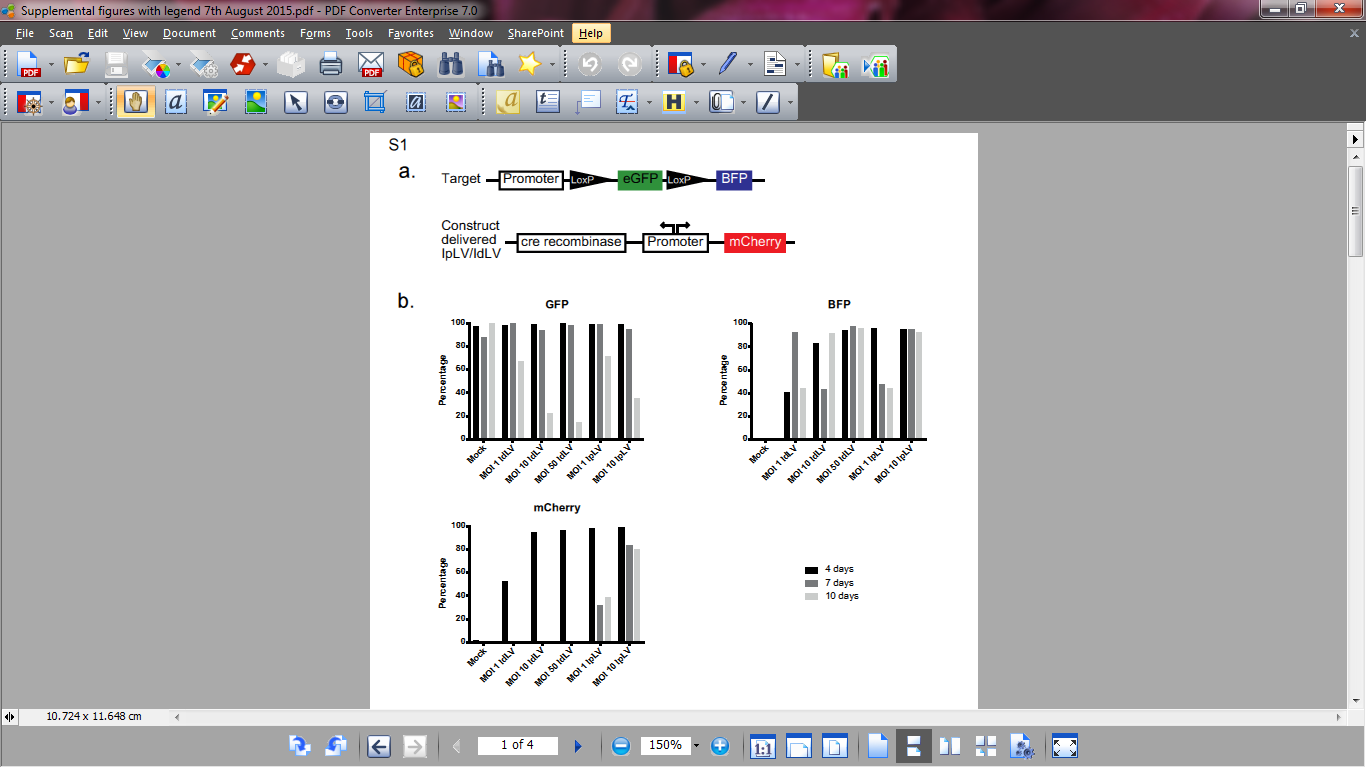


**S2. Longevity of expression from Integration Deficient and proficient Lentiviral Vectors**

To determine the longevity of expression from IDLV and IPLV vectors a K562 cell model was used (a). These cells contained a target locus comprising a green fluorescence reporter gene (eGFP) flanked by *loxP* sites so that expression of cre recombinase in the cells would result in the removal of eGFP and facilitate expression of the adjacent gene, a blue fluorescent reporter (BFP) which was delivered using an SF91-based retroviral vector. IDLV and IPLV vectors containing a bi-directional promoter co-expressing cre recombinase and a red fluorescent protein, mCherry, were used to transduce the K562 target cells (Maetzig *et al*., 2010) which were then grown in liquid culture and the expression level of each reporter gene measured by flow cytometry.

(b) The three panels show flow cytometry measurement of GFP, BFP and mCherry (surrogate for cre) expression in target K562 cells at 4, 7 and 10 days, following transduction with IPLV and IDLV at different multiplicities of infection (MOI). eGFP expression decreases in-line with an increase of BFP production although some co-expression would be expected due to protein longevity. Unlike integrating vectors, IdLV only express cre/mCherry until day 4.

**S3**


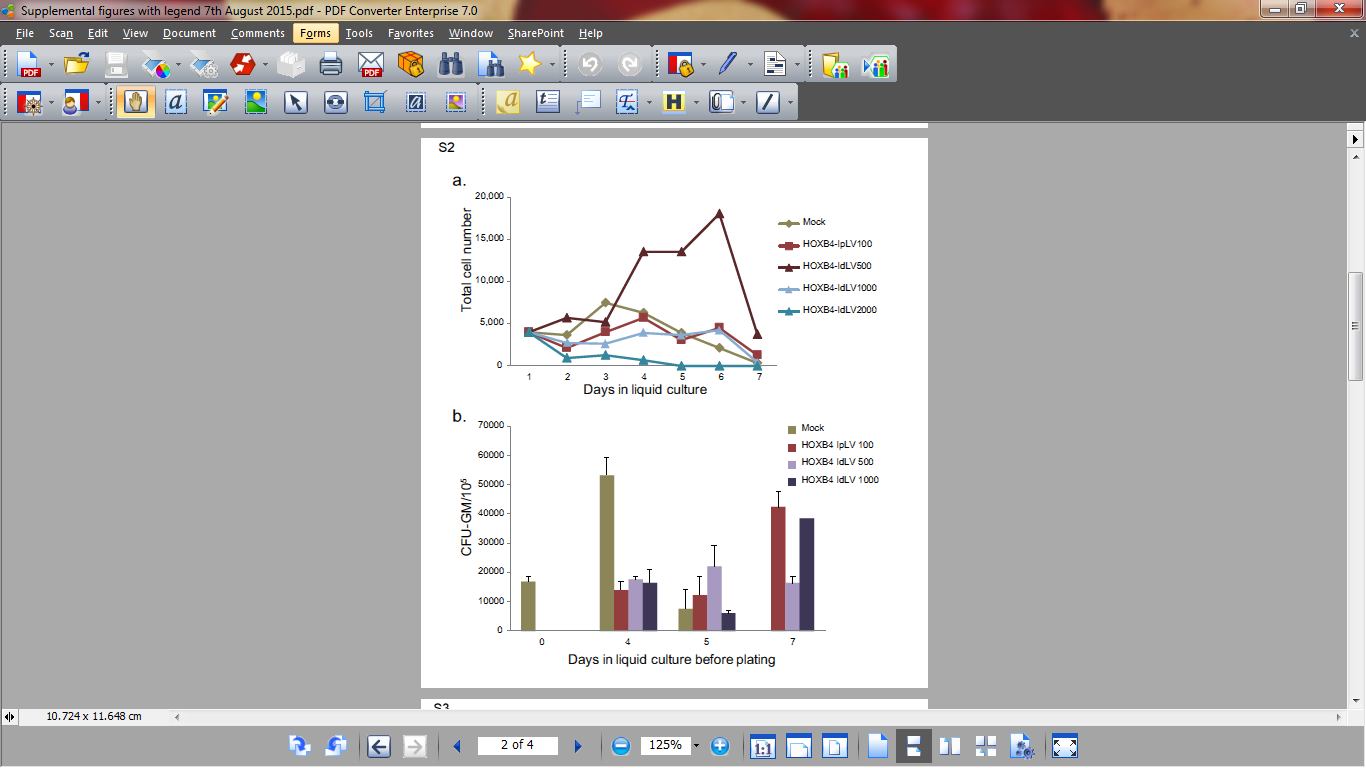


**S3. Protective effects of HOXB4 in *in vitro* clonogenic assays.**

LSK cells were grown in liquid culture and at regular intervals cells were counted and 100 LSKs were removed and seeded in MethoCult GF M3534 semi-solid culture medium (StemCell Technologies), plated in triplicate on 35-mm plastic tissue culture plates and cultured at 37 °C in 5% CO_2_ and fully humidified air. (a) Total cell number in liquid culture was calculated each day over the course of a week. (b) Following 7 days in semi-solid culture, colony numbers were scored. HOXB4 did not necessarily promote replication of LSK cells, but did protect the stem cell phenotype compared to the mock transduced control cells which had reducing clonogenic capacity when grown in liquid culture for a week. Bars show mean ± standard deviation.
